# Ischemic stroke following STA–MCA double bypass

**DOI:** 10.1515/tnsci-2022-0211

**Published:** 2022-02-10

**Authors:** Haijun Zhao, Xiaoguang Tong, Xu Wang, Maohua Ding, Kai Zhang

**Affiliations:** Department of Neurosurgery, Tianjin Huanhu Hospital, Tianjin Neurosurgical Institute, No. 6 Jizhao Road, Tianjin 300350, China; The Fourth Department of Neurosurgery, Tangshan Gongren Hospital, No. 27 Wenhua Road, North District, Tangshan, 063000, China; Department of Interventional Therapy, Tianjin Medical University Cancer Institute and Hospital, National Clinical Research Center for Cancer, Key Laboratory of Cancer Prevention and Therapy, Tianjin's Clinical Research Center for Cancer, Tianjin, China; Clinical College of Neurology, Neurosurgery and Neurorehabilitation, Tianjin Medical University, No. 22 Qixiangtai Road, Tianjin, China

**Keywords:** middle cerebral artery, superficial temporal artery, artery bypass, stroke, cerebral revascularization

## Abstract

**Objectives:**

The surgical technique of STA–MCA double bypass is used to improve blood flow supplied by the distal middle cerebral artery (MCA) to the cerebral territory. This retrospective study from a single center aimed to compare the outcomes following STA–MCA double bypass in 12 patients with recurrent ischemic stroke.

**Materials and methods:**

We retrospectively analyzed the data from patients with internal carotid artery occlusion (ICAO) who had undergone STA–MCA double bypass in our center from January 2016 to December 2020. The surgical indications, evaluation of circle of Willis (CoW), changes in cerebral hemodynamic, surgical results, and follow-up results were analyzed.

**Results:**

Post-operative perfusion-weighted imaging showed hemodynamic improvement in all 12 patients. Ten patients (83.33%) showed clinical improvement, and 2 patients (16.67%) had stable disease. No intracranial infections or acute ischemic events occurred. The post-operative National Institutes of Health Stroke Scale score and modified Barther scores were significantly improved after 180 days of follow-up. Twenty three (96%) anastomoses maintain patency of their bypass vessels, and none had recurrent cerebral infarction during a minimum of 36 months follow-up.

**Conclusion:**

In this small study, in patients with recurrent ischemic stroke without other types of treatment, STA–MCA double bypass surgery was more effective in the subgroup of patients with ICAO and poor blood supply to the CoW and an area of cerebral hypoperfusion that exceeded the area supplied by the MCA.

## 1 Introduction

In patients with internal carotid artery occlusion (ICAO), the main clinical concern is whether the patient develops a recurrent ischemic stroke [1]. Among the various treatment options, the Carotid Artery Occlusion Surgery Study (COSS) has confirmed the superiority of medical treatment [2]. Recently published meta-analysis investigating treatment efficacy in patients with internal carotid artery near occlusion manifested that best medical therapy (BMT) alone is not superior to surgery [3]. Ogawa holds that superficial temporal artery to middle cerebral artery (STA–MCA) bypass benefits patients with symptomatic hemodynamic cerebral ischemia due to occlusive disease [4]. The current evidence-based management guidelines and systematic review concluded that the risk of long-term overall stroke was mildly higher with BMT [5]. Jeffree concludes that patients with carotid artery occlusion and hemodynamic insufficiency have an increased risk of stroke and can benefit from revascularization surgery [6]. Therefore, if a patient with ICAO develops a recurrent ischemic stroke due to compensatory failure of the collateral circulation, a more aggressive treatment approach by the neurosurgeon, including STA–MCA double bypass, may be helpful.

In the present study, we conducted a retrospective analysis of patients with recurrent ischemic stroke without other types of treatment. STA–MCA double bypass surgery was employed in the subgroup of patients with ICAO and poor blood supply to the circle of Willis (CoW) and an area of cerebral hypoperfusion that exceeded the area supplied by the MCA. Therefore, this retrospective study from a single center aimed to compare the outcomes following STA–MCA double bypass in patients with recurrent ischemic stroke.

## 2 Material and methods

### 2.1 Patient population and inclusion criteria

We included patients who underwent STA–MCA double bypass surgery at Tianjin Huanhu Hospital from January 2016 to December 2020. Before surgery, patients were assessed by a neurologist using the National Institutes of Health Stroke Scale (NIHSS) and modified Barther scores to evaluate neurological function. The criteria for inclusion are as follows: (1) patients with ICAO had deteriorating neurological function due to recurrent ischemic stroke and episodes more than twice within 6 months (our treatment began with BMT). (2) Symptoms (ischemic stroke or transient ischemic attack) are due to atherosclerotic ICAO rather than other factors (e.g., cardiogenic embolism). (3) DWI-MRI proved the ischemia in all recurrent events. The ICAO patients with poor blood supply to the CoW and an area of cerebral hypoperfusion exceeded the area supplied by the MCA. (4) Modified Rankin scale ≤ 3; 40–70 years of age (inclusive); and no severe heart disease.

The integrity of CoW has the potential to protect against recurrent ischemic strokes. The CoW score is based on the assumption of the CoW integrity [7]. Conventional angiography evaluates the anterior and posterior communicating arteries and gives each artery a score of 2. A maximum score of 6 indicates a fully intact CoW. If the contralateral intracranial internal carotid artery stenosis exceeded 70%, the anterior and posterior communicating artery scores on the side of the stenosis were considered 0 in this study. This change is necessary because if the contralateral internal carotid artery has severe stenosis (>70%), we consider that the collateral blood flow through the anterior or posterior communicating artery may be compromised. Such changes were also applied in the posterior circulation assessment. If one vertebral artery was occluded and the other vertebral artery had stenosis of >70%, or if the basilar artery had stenosis of >70%, the collateral flow through the posterior communicating artery might be affected. All were considered to have a score of 0 for the posterior communicating artery. Patients with a 0–3 were in the poor CoW integrity group, and those with a 4–6 were in the good CoW integrity group.


**Informed consent:** Informed consent has been obtained from all individuals included in this study.
**Ethical approval:** The research related to human use has been complied with all the relevant national regulations, institutional policies and in accordance with the tenets of the Helsinki Declaration, and has been approved by the Institutional Review Board of Tianjin Huanhu Hospital.

### 2.2 Hemodynamic evaluation

Dynamic susceptibility contrast-enhanced perfusion-weighted magnetic resonance imaging (DSC-PWI) is a relatively newly available tool with a high degree of feasibility and safety that can be used to assess whether a patient needs direct bypass surgery [8]. It also can be used to evaluate the need for direct bypass surgery and evaluate a patient’s long-term prognosis. The DSC-PWI study (repetition time = 2,500 ms, echo time = 80 ms, and flip angle = 90°) was performed preoperatively and postoperatively using a Siemens 3.0T Trio TIM system magnetic resonance imaging (MRI) scanner. The MRI scan included the area from the skull base to the parietal cortex. After scanning, a hydrodynamic injection (3 mL/s) of gadolinium contrast (0.1 mmol/kg body weight) was administered through the antecubital vein. Perfusion source images were obtained using a gradient-echo-echo-planar imaging sequence scanning the whole brain with the following parameters: interlayer spacing = 1.8 mm, layer thickness = 6 mm, and field of view = 230 mm × 230 mm. Perfusion data were processed and converted into parameter maps for cerebral blood volume (CBV), cerebral blood flow (CBF), time to peak (TTP), and mean transit time (MTT).

**Figure 1:**
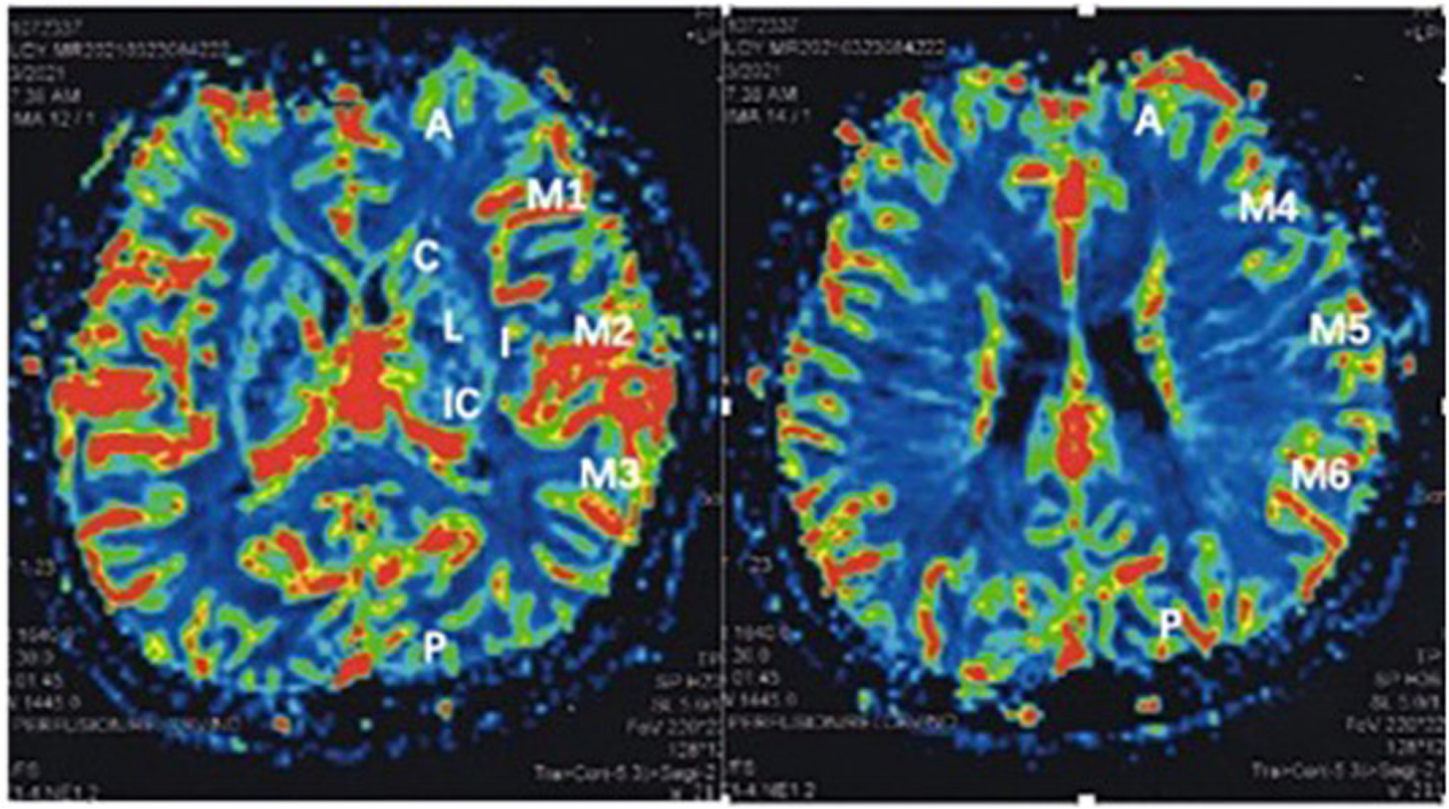
According to the ASPECTS study [9], the MCA supply area is at two standardized regions (basal ganglia and supra-ganglionic levels). The standardized areas are divided into ten sections: C, L, IC, I, M1, M2, and aM3 at the level of basal ganglia area and M4, M5 and, M6 at the level of the supra-ganglionic area. Legend: A, the anterior cerebral artery supplied areas; P, the posterior cerebral artery supplied areas; C, the areas of the caudate nucleus; L, the areas of the lenticular nucleus; IC, the areas of the internal capsule; I, the areas of the insular zone. M1–M6 represent the MCA supplied areas.

The Alberta Stroke Program Early CT Score (ASPECTS) is determined by assessing the MCA supply area at two standardized regions (basal ganglia level and supra-ganglionic level) [9]. Combined with the DSC-PWI, it is an innovative, quantitative, and valuable grading scale for patients with ischemic cerebrovascular disease [8]. The standardized areas are divided into ten sections (Figure 1). The same standardized regional locations were selected for each measurement preoperatively and postoperatively, avoiding infarct locations and large vessel regions compared to the reference regions. The four-color map derived from the DSC-PWI described above can also be scored in this way. CBV-ASPECTS, CBF-ASPECTS, TTP-ASPECTS, and MTT-ASPECTS were measured preoperatively and postoperatively. One score is subtracted if one of the ten MCA supply areas shows perfusion abnormalities. Ten points indicate no ischemic changes in the MCA area, whereas one point indicates a state of severe ischemia and occlusion.

### 2.3 Surgical strategies

**Figure 2:**
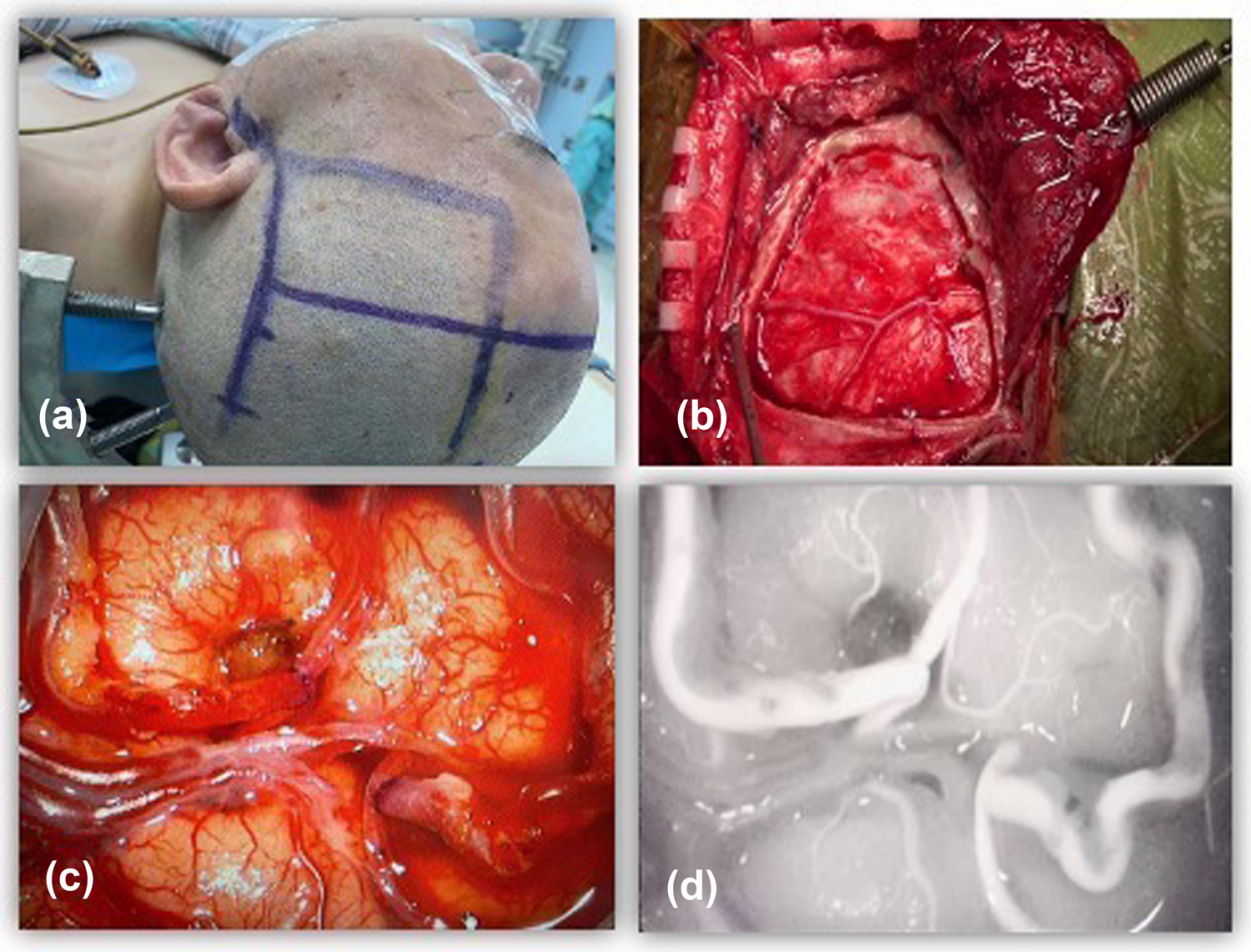
Intraoperative images of the STA–MCA double bypass. (a) The position used and planning for the flap incision. (b) The bone flap and preservation of the middle meningeal artery. (c) Anastomosis between the branches of the STA and the cortical branches of the MCA. (d) Demonstration of patency using indocyanine green angiography.

The superficial temporal artery course was mapped using a Doppler flow detector and separated through a curved incision behind the hairline (Figure 2). The vessels were stripped from the subcutaneous tissue under the microscope. We use a combination of bipolar cautery and scissor-dilated dissection to access the superficial temporal artery from the connective tissue attachment and separate small branches from the main trunk. When the trunk and two branches of the superficial temporal artery were separated, they were flushed with heparinized saline, cut distally, and protected with poppy-impregnated cotton.

The donor artery is prepared by removing the connective tissue distal to the donor’s vessel. The end of the donor artery was increased with an oblique 45–60° dissection to increase vascular anastomosis. The caliber on the selected recipient vessel was similar to that of the superficial temporal artery. A white latex glove piece was placed between the recipient artery and the adjacent brain surface to protect the brain tissue and optimize the field of view. After cross-clamping the recipient vessel with an aneurysm-blocking clip, the recipient’s vessel was dissected with a T-shaped incision, and the arterial lumen was flushed with heparinized saline. Each end-lateral anastomosis was performed separately, with each superficial temporal artery donor being anastomosed to each MCA recipient using a double 10-0 nylon interrupted suture technique. Each anastomosis was blocked for 20–30 min. Indocyanine green (ICG) angiography was performed inter-operatively (Zeiss Pentero Flow 800 microscope; Zeiss Corporation, Oberkochen, Germany/ICG dye; Buffalo Grove, Illinois [Akorn]).

### 2.4 Postoperative management

Patients undergoing STA–MCA double bypass surgery were treated with the BMT before surgery. Our treatment optimized all medical therapeutic options, including judicious blood pressure control, aggressive lipid lowering with statins, glycemic control, and smoking cessation. One hundred milligrams of aspirin per day were prescribed for 1 year. The patients received CT angiography (CTA) to assess graft patency. Postoperative angiography and DSC-PWI were performed to evaluate the hemodynamic improvement. All patients were followed up in our outpatient clinic for at least 36 months. Patients’ neurological function was scored on the NIHSS and statistically analyzed. Patency of the bypass and changes in collateral vessels were assessed by CTA or digital subtraction angiography (DSA).

### 2.5 Statistical analysis

Fisher’s exact probability method is used for statistical tests of count data. The statistical test for measurement data was *t*-test or ANOVA if they conformed to a normal distribution; the rank-sum test was used if they did not conform to a normal distribution. The test level *α* was set at 0.05. IBM SPSS Statistics for Windows, version 27.0 (IBM Corp, Armonk, NY, USA) and R software version 4.0.3 (R Core Team, 2014) were used for statistical analysis and graphing.

## 3 Results

### 3.1 Perioperative consequences and complications

According to the inclusion criteria, 12 ICAO patients were treated with STA–MCA double bypass surgery (Table 1). The patency of each anastomotic stoma was confirmed by intraoperative ICG injection. The complication was subdural hemorrhage in one patient (out of ten patients) in the operative area and received conservative therapy (Table 2). No intracranial infections or acute ischemic events occurred. No patient in our cohort suffered epileptic seizures; patients were given regular anti-epileptic treatment after surgery, even when the operated area was limited to the cortex.

**Table 1:** Patient characteristics

Pt. no.	Sex/age (years)	Symptoms	Episodes ≤6 months	Side of ICAO	The score of CoW	NIHSS score	Modified Barther scores
1	FM/64	Limb weakness (R), partial motor aphasia, and dysarthria	3	Left	1	9	32
2	M/58	Limb weakness (R) and partial motor aphasia	3	Left	3	6	45
3	M/66	Limb weakness (R) and partial motor aphasia	3	Left	2	6	45
4	M/63	Limb weakness (R) and partial motor aphasia	3	Left	2	7	40
5	M/45	Limb weakness (R), partial motor aphasia, and dysarthria	4	Left	2	8	34
6	M/45	Limb weakness (L), dysarthria, and facial paralysis (L)	4	Right	3	7	38
7	FM/53	Limb weakness (R), partial motor aphasia, dysarthria, and facial paralysis (R)	4	Left	2	9	30
8	M/55	Limb weakness (R)	2	Left	4	5	52
9	M/52	Limb weakness (R), partial motor aphasia, dysarthria, and facial paralysis (R)	2	Left	3	6	40
10	FM/56	Limb weakness (L), dysarthria, and facial paralysis (L)	3	Right	2	7	42
11	FM/58	Limb weakness (L) and dysarthria,	2	Right	3	5	55
12	FM/55	Limb weakness (L) and facial paralysis (L)	3	Right	4	5	60

Pt. no., patient number; ICAO, internal carotid artery occlusion; NIHSS, National Institute of Health stroke scale; M, male; F, female. CoW, circle of Willis.

**Table 2:** Follow-up information for patients with ICAO after STA–MCA double bypass

Pt. no.	Complications	Follow-up period (months)	Ischemic events <30 days	Ischemic events >30 days	Graft patency on 180 days	NIHSS score for 180 days	Modified Barther scores for 180 days
1	None	36	None	TIA	Yes	6	75
2	None	42	None	None	Yes	4	90
3	None	38	None	None	Yes	4	90
4	None	41	None	None	Yes	5	80
5	None	48	None	None	Yes	5	85
6	None	40	None	TIA	No	4	90
7	None	45	TIA	None	Yes	6	75
8	None	43	None	None	Yes	3	100
9	None	42	None	None	Yes	4	85
10	Subdural hemorrhage	40	None	None	Yes	5	70
11	None	42	None	None	Yes	4	75
12	None	38	None	None	Yes	5	65

Pt. no., patient number; NIHSS, National Institute of Health stroke scale; TIA, transient ischemia attack.

### 3.2 Improving neurological function

NIHSS scores and modified Barther scores were calculated for all patients perioperatively and in follow-up. The mean NIHSS score was 6.50 [5.75; 7.25] preoperatively. During follow-up, the mean NIHSS score was 4.50 [4.00; 5.00]. Statistical analysis revealed statistically significant differences in the NIHSS scores measured in the preoperative and follow-up periods (*P* < 0.005). The mean modified Barther score was significantly improved from 41.00 [35.00; 50.25] preoperatively to 82.50 [75.00; 90.00] at the last follow-up (*P* < 0.005). Whether a patient received any benefit from the surgery depended on their preoperative neurological status. Those with higher initial NIHSS scores and lower initial modified Barther scores experienced more significant improvements in their NIHSS scores and modified Barther scores after surgery.

Ten (83.3%) of the 12 patients who had a history of ischemic stroke with limb weakness showed improvement in muscle strength. Eight (80.0%) of the 10 patients who had residual neurological deficits with partial motor aphasia and/or dysarthria showed improved neurological function or language function progress. All these patients had improved neurological function or decreased occurrence of transient ischemia attack (TIA) during the follow-up period.

### 3.3 Bypass patency

**Figure 3:**
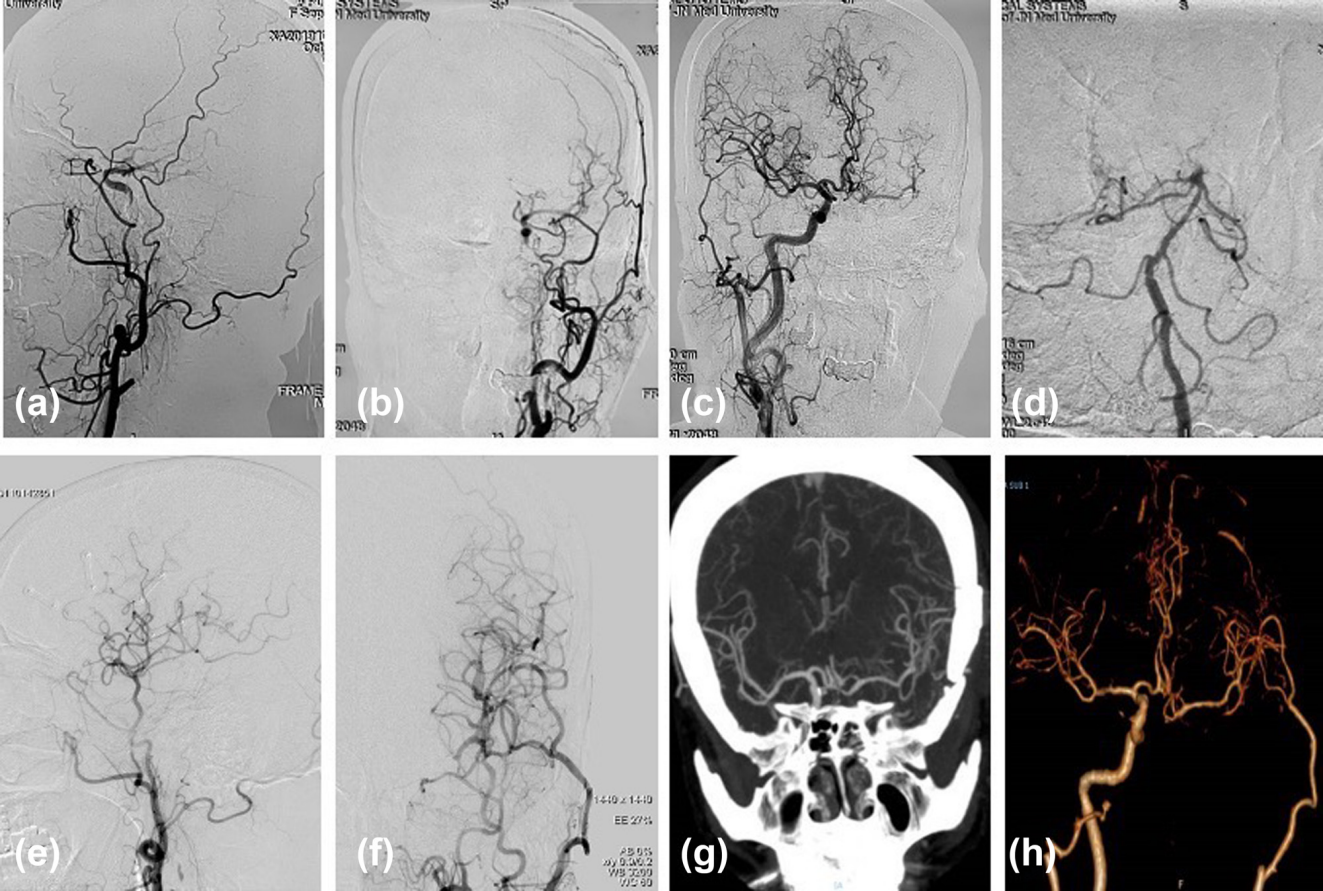
Patient with left ICAO. DSA imaging before (a–d) and after (e–f) STA–MCA double bypass. CTA images (g–h) were obtained 1 year after the bypass surgery.

One week after surgery, CTA revealed a 100% patency rate for the anastomotic stomas. Twelve patients (24 anastomoses in total) underwent DSA of 6 vessels for at least 6 months after surgery to determine the surgically induced collateral circulation status (Figure 3). All operated hemispheres were predominantly supplied by the external carotid artery system in this series. Twenty three (96%) anastomoses remained patent, and circulating blood flow entered the MCA region. One patient had occlusion of a bypass vessel at the 1 year follow-up evaluation. No new infarct foci were seen on the MRI scan, and the patient had no new neurological deficits.

### 3.4 Hemodynamic changes in the operated side

**Figure 4:**
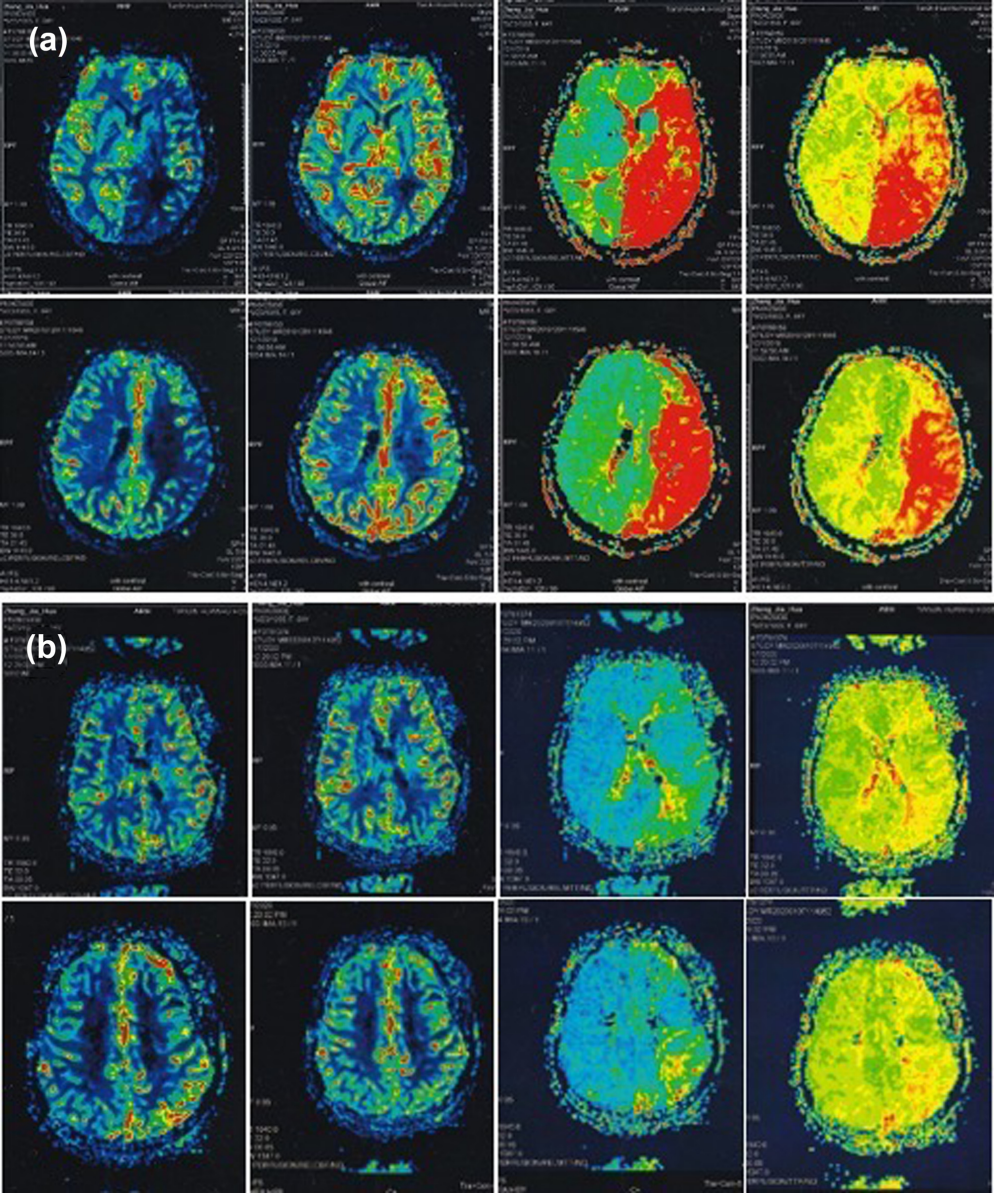
A 64-year-old woman with recurrent ischemic stroke. Cerebral perfusion imaging before (a) and after (b) STA–MCA double bypass.

Table 3 summarizes the analysis of PWI-ASPECTS score data before and after bypass surgery. Postoperative DSC-PWI showed hemodynamic improvement in all 12 patients (Figure 4). The CBF-ASPECTS increased from 5.00 [5.00; 6.00] to 8.00 [8.00; 9.00], the MTT-ASPECTS increased from 2.00 [2.00; 3.00] to 7.00 [6.00; 8.00] and TTP-ASPECTS increased from 3.00 [3.00; 3.00] to 6.00 [5.00; 8.00] postoperatively, with a statistically significant difference. However, the CBV-ASPECTS did not significantly differ before and after the bypass surgery. These changes in PWI-ASPECTS score parameters after direct hematopoietic reconstruction suggest that significant improvements in cerebral perfusion in the surgical hemisphere can be expected after surgery. The patients with lower preoperative PWI-ASPECTS score have more significantly improved postoperative cerebral perfusion.

**Table 3:** The PWI-ASPECTS scores before and after STA–MCA double bypass

	Pre-operation	Post-operation	*p*-value
CBF-ASPECTS	5.00 [5.00; 6.00]	8.00 [8.00; 9.00]	0.014
CBV-ASPECTS	9.00 [8.00; 9.00]	9.00 [9.00; 9.00]	0.058
MTT-ASPECTS	2.00 [2.00; 3.00]	7.00 [6.00; 8.00]	0.009
TTP-ASPECTS	3.00 [3.00; 3.00]	6.00 [5.00; 8.00]	0.009
NIHSS score	6.50 [5.75; 7.25]	4.50 [4.00; 5.00]	0.003
Modified Barther scores	41.00 [35.00; 50.25]	82.50 [75.00; 90.00]	0.002

Note: paired rank-sum test applied, statistical description as median [25% spacing; 75% spacing].

## 4 Discussion

In the COSS trial scheme, 40% of the stroke incidence in the drug group was set based on previous studies. Due to the improvement in drug treatment, the incidence of strokes significantly decreased, resulting in the deviation from the original study design. In the current evidence-based management guidelines and systematic review, STA–MCA bypass and BMT were associated with similar rates of a composite of long-term stroke [5]. However, the total patient cohort consisted of 2,419 patients, of which 1,122 patients come from the International Study of Extracranial-to-Intracranial Arterial Anastomosis (EC–IC bypass trial). This trial included the inability to identify and separately analyze a subgroup of patients with impaired cerebral hemodynamics due to occlusive cerebrovascular disease in whom surgical revascularization might be more beneficial [10]. Several studies have suggested that STA–MCA bypass in carefully selected patients helps improve cerebral hemodynamic parameters as well as the secondary prevention of symptomatic secondary cerebral ischemic events [11,12]. This was concurred by a systematic review which found that patients with severe hemodynamic failure secondary to atheroocclusive disease appear to benefit from direct EC–IC bypass surgery [13]. At present, each patient should receive more precise treatment by reasonably assessing the individual differences of each patient to reduce the recurrence rate of stroke.

In patients with cerebral hemodynamic impairment and poorly compensated collateral circulation in ICAO, the annual risk of stroke recurrence is 9–18% [14,15]. Evaluating the collateral circulation is clinically vital in interpreting clinical manifestations, formulating treatment plans, evaluating treatment outcomes, and determining prognosis [16,17,18]. Cerebral infarcts are less likely in patients with carotid occlusion and collateral supply via the CoW than those with leptomeningeal or ophthalmic collaterals [19,20]. Our study used a CoW score system to evaluate CoW integrity. The score of CoW may be a prognostic factor for failure of drug therapy in these cases. Most of the patients in the present series had poor CoW collaterals, suggesting that these patients are unlikely to have a spontaneous improvement in cerebral hemodynamics. As conventional multi-branch angiography does not apply to all patients, a method that combines angiographic information with non-invasive perfusion data will significantly contribute to our understanding of the collateral circulation. The area of cerebral hypoperfusion that exceeded the area supplied by the MCA is another implication of impairment cerebrovascular reserve. In patients with strokes who have intracranial and extracranial artery stenosis or occlusions, poor blood flow can be caused by abnormal hemodynamics (impaired autoregulation) beyond the lesion [21]. The patients in our present study suffered relevant (≥70%) stenosis of multiple vascular segments in addition to unilateral ICA alone and had a recurrent ischemic stroke. It is important to note that this subgroup has unmet hemodynamic needs and a greater ischemic burden than COSS and explains why clinical studies on bypass surgery for atherosclerotic cerebrovascular disease have become popular again [22,23,24].

In this study, cerebral hemodynamics was markedly improved in 12 patients (100%). Moreover, after analyzing the PWI-ASPECTS score data before and after bypass surgery, the patients with lower preoperative PWI-ASPECTS score have more significantly improved postoperative cerebral perfusion. According to this study, the more severe the reduction in cerebral hemodynamics before STA–MCA bypass, the greater the improvement in cerebral hemodynamics after STA–MCA bypass. We speculated that a more severe reduction in blood perfusion could cause a higher requirement of the brain tissue for blood. Hemodynamics will significantly improve once fresh extracranial blood reaches the brain tissue. Therefore, this finding confirms that reducing preoperative cerebral perfusion is vital to preventing ischemic stroke in patients undergoing EC–IC bypass.

As highlighted by COSS, the incidence of perioperative complications significantly impacts prognosis [25]. Some surgeons with a 2-day training or less than 10 bypass operations were also admitted to the COSS, which could lead to an abnormal rise in perioperative adverse events. Since 2005, we have performed over 1,000 different types of bypass surgery, so we have extensive experience in bypass surgery. Before performing the procedure, the depth of the anastomosis, the size of the orifice, and the difference in diameter between the donor and recipient vessels should be considered. Concerning suturing techniques, interrupted sutures can effectively improve the patency of the anastomosis and reduce the stenosis of the mouth. A fair distribution of sutures is also necessary. In this series, there was one bypass vessel occlusion at 1 year follow-up. However, the patient had no new neurological deficits. This may be related to the difference in pressure between the two bypass vessels. The STA–MCA bypass double anastomosis balances the competing relationship between the two vessels of the superficial temporal artery. It introduces a higher total blood flow over the surface of multiple hypoperfused areas in a distributed flow pattern. Although STA–MCA bypass over perfusion has been reported, no cases of postoperative cerebral hemorrhage were seen in this study [26]. In our series, the terminal branch of the superficial temporal artery was anastomosed to the cortex artery of MCA, anastomosing to separate superior and inferior MCA branches. This may allow for “load sharing” of higher total bypass flow across separate areas of hypoperfusion. Although more research is needed, based on the results of the EC-IC bypass trial and the single STA–MCA bypass in the COSS, a double bypass may represent a relevant consideration for reducing the risk of ipsilateral stroke recurrence in appropriately selected patients [27,28].

There were some limitations to our study. First, the major limitation of our study is the single-site, single-surgeon nature of the project. However, these factors eliminate variations in technique that a multicenter study with multiple surgeons may introduce. Second, considering the convenience, economic burden, and radiation exposure, we prefer DCS-PWI to evaluate the changes in cerebral blood flow. However, this measure is only a relative indicator comparing the bilateral cerebral hemispheres. To understand the absolute changes in the metabolic index of the ipsilateral hemisphere, the use of the regional oxygen extraction fraction might be better because the latter is more precise.

Furthermore, the number of patients included in this study may be relatively small. However, they are sufficient in offering preliminary data to show the effectiveness of bypass surgery for those specific populations. In the future, a more extensive study with an elaborate design may be needed to determine these factors.

## 5 Conclusion

In this small study, in patients with recurrent ischemic stroke without other types of treatment, STA–MCA double bypass surgery was more effective in the subgroup of patients with ICAO and poor blood supply to the CoW and an area of cerebral hypoperfusion that exceeded the area supplied by the MCA.
